# Comparison of techniques to control the aggressive environmental invasive species *Galenia pubescens* in a degraded grassland reserve, Victoria, Australia

**DOI:** 10.1371/journal.pone.0203653

**Published:** 2018-11-14

**Authors:** Ako H. Mahmood, Singarayer Florentine, Friedrich P. Graz, Christopher Turville, Grant Palmer, James Sillitoe, David McLaren

**Affiliations:** 1 Centre for Environmental Management, Faculty of Science and Technology, Federation University, Mount Helen, Victoria, Australia; 2 Central Queensland University, School of Health, Medical and Applied Sciences, Bruce Highway, North Rockhampton, Queensland, Australia; 3 Department of Economic Development, Jobs, Transport, and Resources, Victorian and La Trobe University, AgriBiosciences Centre, Bundoora, Australia; USDA-ARS Fort Keogh Livestock and Range Research Laboratory, UNITED STATES

## Abstract

Across many southern regions of Australia, native grasslands have become seriously threatened by human activity, with only a fraction of the original areas remaining undisturbed. In particular, the introduction and establishment of exotic invasive weeds has caused significant degradation to the ecosystems in these areas by contributing to a decrease in native plant density and diversity, and this has ultimately led to major changes to the ecosystem structure and function. One such example is *Galenia pubescens*. Our objective of this study was to assess the effectiveness of four different attempts to control *G*. *pubescens*: herbicide control with glyphosate; organic herbicide control with pine oil; the application of mulch; and the addition of seeds of native species to the seedbank. Results shows that any one single control strategy is insufficient to control *G*. *pubescens*, and, in addition, it has shown that regeneration of native vegetation is limited unless direct seeding is applied. There was a strong indication that a combined strategy employing more than two of the aforementioned techniques is likely to be the most effective approach, at least in the short term. Underscoring the complexity of this task, our analysis on foliage cover of *G*. *pubescens* shows that the interaction of pine oil and glyphosate treatments appeared to be very effective after six months, but were not so effective after 18 months. By contrast, seeding with native seeds was not particularly effective at six months, but its longer-term contribution appears to be effective at 18 months. Further, our results obtained from the seedbank abundance study indicate that time alone was not a significant factor in restoration of the grasslands (p = 0.165); however there were interactions with time, shown by time*glyphosate (p = 0.008) and time*seeding (p = 0.016). Both interactions indicated that the applications of glyphosate and seeding were more beneficial after 18 months compared to six months. However, full regeneration of invaded native grasslands may not be possible unless further restoration programs are re-implemented after the first cycle of *G*. *pubescens*’ treatments have been completed.

## Introduction

Destruction of native grasslands in Australian territories has expanded [1], and in particular in the state of Victoria, lowland native grassland is perceived as one of the state's most endangered vegetation communities [2,3,4]. It is listed as a ‘threatened entity’ under Victorian legislation, and has been nominated as a ‘critically endangered community’ under Australian Federal legislation. In western Melbourne, 1670 ha (23%) of the 7230 ha of native grassland present in 1985 has been damaged by development, and a further 1469 ha (21%) was degraded to non-native grassland by 2000. In the remaining areas of native grassland, fewer patches, and greater distances between patches, were recorded in 2000 than in 1985, showing that, in addition to the initial invasion problem, fragmentation of these native areas has intensified [1].

Concern about the loss of temperate native grasslands in Victoria is not new. For example, in 1916, Sutton [5] reported that the grasslands west of Melbourne, while not being favoured for residential purposes and not being much built over, had been devoted so completely to pastoral and farming uses, that barely any part remained in a virgin condition. However, since the 1980s, Melbourne has experienced a period of rapid residential and industrial development, due to a substantial increase in population growth. Of particular concern here is that in addition to the obvious threats posed by development of housing and related infrastructure, native grasslands of this region are being significantly degraded due to aggressive and rapid exotic weed invasion [6].

In this latter respect, various factors appear to give non-indigenous species a competitive advantage over indigenous grassland species. These include the disbursement of supplement-rich water from neighboring farming or urban areas, the soil-unsettling practices connected with vehicle and stock access, as well as the infrastructural activities of road and rail management agencies and of utility companies [7]. Invasive species are also favoured by inappropriate land management [8,9], particularly the failure to remove biomass by systematic burning or grazing. This leads to an overabundance of dead plant material, whose consequent decomposition releases nutrients that further support weed expansion [8]. Unless different management practices and innovative control mechanisms are instituted, the remaining grasslands will be increasingly threatened by weed invasion and other degradation processes. Indeed, it has been widely noted that many of these highly invasive alien species, particularly *Nassella neesiana* (Trin. & Rupr. Barkworth) and *G*. *pubescens*, now present the major threat to Victorian native grasslands [10,11,12].

The options available to control *G*. *pubescens* infestation in Australia are currently restricted to herbicide treatments and mechanical removal [13], which limits the success of control options in many situations. However, compounding the problem is that studies have shown that increasing use and reliance on a few synthetic herbicides has not only resulted in environmental and human health issues, it is leading to an expanding incidence of herbicidal resistance among many weed species [14]. Furthermore, options for chemical weed control in non-arable situations are limited to either very expensive spot treatments, or blanket coverage which is non-selective, and such intensive use of synthetic herbicides can result in significant soil and groundwater contamination. In addition, Warnock et al. [15] suggested that in general, herbicide application was only effective in the short-term, since the bare soil areas resulting from the herbicide treatments actually assists in the promotion of new seedlings which arise from pre-existing seedbanks laid down in previous years. Indeed, several authors suggest that this phenomenon is common to many weed species, and that herbicide treatment aimed at achieving short-term goals can result not only in reinvasion, but can also increase weed abundance to a level greater than that existed prior to treatment [16]. Clearly, this unanticipated outcome moves the composition of the vegetation community further away from the desired outcome [17, 18]. In addition, use of herbicide as a control method may have several possible side-effects, including spray drift from aerial application which can impact surrounding areas [19]. It has also been observed that any areas that are physically missed in a particular herbicide application exercise, may represent a constant source of seed for rapid re-colonisation, and a significant complicating factor is that some herbicides, such as glyphosate, are not species-specific, therefore, non-target species are likely to be inadvertently affected [20].

It has also been noted that there are several disadvantages associated with mechanical control methods. They may not always be applied when required, particularly where the ground is too wet, steep or stony for machinery to operate successfully, or perhaps the area of infestation may be too large to be covered in a reasonable time [21]. Also, in situations where machinery can access the site, its use may damage fragile soil structures, stimulate further weed emergence from the seed bank by exposing stored seeds, and encourage erosion by leaving wheel tracks.

Of interest to this investigation is that, according to a recent report from the Weed Society of Victoria, there are no biological control options currently available for *G*. *pubescens* in Australia [22]. Whilst one study to investigate options for chemical control of this species has recently been undertaken [13], at this time there are no known organic control agents available. Therefore, efforts to develop effective alternative means of weed control are required that are not only eco-friendly, but are also cost effective and biologically robust.

This study aims to provide evidence to inform long-term management solutions intended to swing the competitive advantage towards native grasslands species by: (i) selectively killing mature *G*. *pubescens* plants through spot applications of glyphosate; (ii) reducing the *G*. *pubescens* seedbank through application of an essential oil (pine oil); (iii) applying mulch (sugar and sawdust) to increase microbial drawdown of soil nitrogen to reduce competitiveness of nitrogen-loving colonising weeds such as *G*. *pubescens;* and (iv) introducing native grassland species to colonise bare ground created by the herbicide, pine oil and mulch treatments to provide adequate vegetation competition as a means for reducing reestablishment of *G*. *pubescens*. If these techniques are effective, new eco-friendly rehabilitation and management methodologies may be developed to accelerate restoration efforts in these degraded grasslands, and innovative seedbank control techniques, such as that proposed in this investigation, may become an effective tool for eradication and control of other priority weed species in the future.

## Materials and methods

### Study site

The study was conducted in an area proposed for addition to the Western Grassland Reserves, approximately 12 km from Werribee, at 37° 49' 5.63" S and 144° 34' 58.77" E and an altitude of 66 m. The site is representative of the distribution of *G*. *pubescens* southeast of the Werribee River, Victoria. Prior to this experiment in 2013, soil data was collected under high density *G*. *pubescens’* infestations, and at this time it was noted that soils with high cover generally had higher potassium and sulphur levels, as well as a low pH. For use in the treatments, pine oil, white sugar and sawdust were obtained from commercial trade shops. We obtained permission from Parks Victoria, Australia to carry out this project in the Western Grassland Reserves.

### Target species: *Galenia pubescens*

*Galenia pubescens* (Eckl. & Zeyh.) is a prostrate perennial herb indigenous to South Africa [23]. It is a member of the Aizoaceae family (Ice plants) and is a C_4_ plant that has adapted to grow in the deserts of southern Africa [24]. It is commonly known as ‘blanket weed’, ‘carpet weed’ or ‘green galenia’ and is considered a serious environmental weed in many countries including Australia, where it invades dry coastal vegetation, lowland grassland and grassy woodland, dry sclerophyll forest and woodland, and rocky outcrop vegetation [25].

*Galenia pubescens* was first reported in Victoria in the early 1900s [22] and has since spread through most southern states in Australia [26], with infestations being particularly severe in the upper Hunter region of New South Wales [13]. *G*. *pubescens* forms a roughly circular mat on the ground, growing out from a deep central stem in a similar habit to *Polygonum aviculare* (wireweed) [13]. *G*. *pubescens* has been found to smother other plants, and infestations have been linked with statistically-significant lower levels of native species richness and diversity [27]. Its smothering habit of growing over surrounding vegetation leads to its dominance in many instances.

At the local level, infestations of *G*. *pubescens* are becoming an increasing issue within the Western Grassland Reserve where it dominates areas previously occupied by native grassland species. *G*. *pubescens* is a drought-tolerant, salt-tolerant herbaceous plant, and, importantly, it has been found to have a higher extractable nitrate reductase activity than for any previously observed higher plant species [28]. In 1979, Williams [29] showed that consequent high levels of nitrates and soluble oxalates produced by *G*. *pubescens* can be toxic to stock. Williams [29] also showed that when *G*. *pubescens* grows on infertile soils, these chemicals were not found to be produced in high levels, but when grown in fertile soils these chemicals can accumulate to toxic levels.

### Experimental design and treatments

This study was carried out in four 96 m x 96 m study-blocks. Each block was partitioned into 6 m x 6 m subplots, giving 16 plots with almost uniform infestation levels of *G*. *pubescens*. The trial thus consisted of 16 treatments, conducted in a completely randomized block-design (CRBD) which was replicated four times across the study blocks. The five basic treatments were: (i) control (no treatment); (ii) a chemical herbicide (glyphosate); (iii) essential oil (pine oil); (iv) mulch (sawdust + white sugar), and (v) seed addition (a mixture of native grass seeds). Each of these was applied as separate treatments, and as all possible combinations of treatments: (vi) glyphosate + pine oil, (vii) glyphosate + mulch, (viii) glyphosate + seeding, (ix) pine oil + mulch, (x) pine oil + seeding, (xi) mulch + seeding, (xii) glyphosate + pine oil + mulch, (xiii) glyphosate + pine oil + seeding, (xiv) glyphosate + mulch + seeding, (xv) pine oil + mulch + seeding, (xvi) glyphosate + pine oil + mulch + seeding. Each treatment plot was permanently marked with numbered fence droppers, individually labelled.

### Application of treatments

#### Glyphosate application

A new generation of glyphosate herbicide, Raze® (Sipcam Pacific Australia Pty. Ltd.), was used for the glyphosate application treatments. It is a water-soluble liquid, from the glycine group of herbicides. It is a non-selective, broad-spectrum, systemic herbicide that is used for control of a wide range of annual and perennial weeds [30]. The herbicide is absorbed by plant leaves and green stems, and is then translocated through the plant to the root system. The active ingredient inhibits a plant enzyme (5-enolpyruvylshikimate-3-phosphate [EPSP] synthase), causing a breakdown in the metabolic photosynthesis pathway, which leads to the eventual death of the plant [31]. The herbicide was applied following the appropriate herbicide treatment combinations, to all but the control plots, in early March 2014. The rate used was 90 ml of herbicide per 10 L of water. Plots receiving selective glyphosate treatment were sprayed using a backpack sprayer (Solo®, model 425) at 90 psi, with a piston pump and flat spray nozzle). The entire surface area of the plots were sprayed. The ambient temperature during application was 30°C, and there was no wind.

#### Pine oil application

Pine oil, which is an essential oil, was selected for this research because it is widely commercially available. Pine oil (BioWeed™ and BioSeed™) is a patented distilled essential oil of radiata pine (Pinus radiata), produced by Certified Organics Pty Ltd, which is a joint Australian/New Zealand company. BioWeed™ and BioSeed™ herbicides are not systemic herbicides. They work by stripping the outer coating of plant and seed material when it comes into contact with the preparation, causing massive cell collapse and desiccation. A solution of 10% pine oil in water was applied, with a watering can, at a rate of 72 L per 36m^2^. The entire surface area of each of the selected plots was treated in April 2014, immediately after rainfall, in order to avoid any significant evaporation effect.

#### Mulch treatment

A mulch, consisting of white sugar (sucrose) and sawdust, was added to the soil to increase microbial populations and CO_2_ production, while also contributing to a reduction in soil nitrogen [32, 33]. Each of the selected plots was treated by evenly spreading 12.5 kg of white sugar over the entire plot, followed by the addition of 20 kg of sawdust, to a depth of approximately 5 cm over the soil surface.

#### Seed addition treatment

A seed mix of four native grass species was spread evenly onto the surface of each of the plots prior to a mulch treatment in order to provide competition to *G*. *pubescens*. Prior to applying the seeds, plots were lightly harrowed using a metal rake to aid seed penetration into the soil, and the seeds were hand-broadcast in an even fashion. The seed mix used in this experiment comprised a mixture of *Rytidosperma acerosum* (Vickery) seed at a rate of 20–30 kg/ha, *Bothrichloa macra* (Steud.) (red-leg grass) seed at 30–40 kg/ha, *Themeda triandra* (Forsk.) (Kangaroo grass) seed at 40–50 kg/ha and *Austrostipa bigeniculata* (Hughes) (spear grass) seed at 20–30 kg/ha. The seed was sourced from Flora Victoria, a registered producer of native seed.

#### Combined treatment applications

The timing of all combined treatments was chosen to permit each of them to achieve their optimal effect, as follows. In the plots which received glyphosate plus any other treatment, glyphosate was applied first. The pine oil was applied two weeks after glyphosate, then the mulch was added two more weeks after the pine oil treatment (four weeks after glyphosate). Native seeds were scattered onto designated plots one week after the mulch application (five weeks after glyphosate). However, where the seeding treatments were combined with mulch, the addition of mulch occurred after seed addition in order to protect seeds and to provide warmth to the soil.

In the two combination treatments, glyphosate was combined with pine oil (vi), mulch (vii) and seeding (viii), pine oil was combined with mulch, (ix), and seeding (x), and mulch was combined with seeds (xi). In the three combination treatments, glyphosate was applied with pine oil and mulch (xii), pine oil and seeding (xiii), mulch and seeding (xiv) and pine oil was applied with mulch and seeding (xv). In the four combination treatment, all treatments were combined; glyphosate, pine oil, mulch and seeding (xvi). All treatments were completed by early- to mid-May 2014.

#### Measurements

Prior to the application of treatments in autumn 2014, the foliage cover of the plant community was assessed within each of the treatment plots. These community assessments formed a baseline against which post-treatment community compositions could be compared. The response of the plant communities within each treatment plot was determined for both the above and below-ground communities as outlined in the following sections.

#### Vegetation survey

To measure the effectiveness of the treatments, three surveys of the aboveground vegetation cover were carried out. The baseline survey was conducted in October 2013, prior to commencement of the experiment. Following the application of all treatments in autumn 2014, there were two follow-up assessments, the first after six months in the spring of 2014 and the second after 18 months in the spring of 2015. The procedure for measuring the aboveground vegetation is as described in Dix [34], Mueller-Dombois and Ellenberg [35], and Rodriguez and Jacobob [36]. Within each 6 m x 6 m plot, four 5 m transects were arranged at 1 m intervals across each plot. To avoid possible interference from ‘edge effects’, the first transect line was set out 1 m from the corner posts. Using the point intercept technique, measurements were taken every 20 cm for each transect. This resulted in 25 intercept points per transect and 100 intercept points for each plot. At each intercept point, a record was made of whether the ground at that point was: bare ground or rock, covered with litter, or covered by vegetation. Where covered by vegetation, the plant species were recorded. The point intercept method was also used to characterise percentage cover by species; that is, at each of 100 intercept points within the 64 6 x 6 m plots, a record was made of the species present. This provided a mean percentage score over all found species across all plots.

#### Seedbank sampling and identification procedure

To determine the effect of the treatments on seedbank composition, soil samples were collected from all plots prior to treatments being applied, and the constituents of the soil seedbank were recorded. This data was subsequently used as a baseline for a comparison of the effectiveness of single treatments, as well as combinations of treatments. During the remainder of the experiment, four soil samples were taken from all plots at two time intervals, six months (October 2014) and 18 months (October 2015) after treatments were applied. The procedure for measuring the composition percentage of the soil seedbank is described in Bertiller [37] and Graham et al. [38].

#### Statistical analyses

Cover percentage and seedbank data were analysed using Repeated Measures ANCOVA to test the four treatments (glyphosate, pine oil, seeding and mulch) and their associated interactions over two time periods (six months and 18 months after treatment). Significant interactions (up to three way interactions) were investigated using interaction plots and investigation of the simple effects. The initial time of treatment cover percent and seed abundance were used as a covariate in their respective analyses. The statistical analyses assumptions were investigated by checking univariate and multivariate normality of the residuals as well as multivariate outliers.

## Results

### *Galenia pubescens* foliage cover percentage

The assumption checking indicated that the repeated measures analysis of cover percent was appropriate. The initial cover percent was used as a covariate and was not significant (p = 0.200). There was a significant time effect (p = 0.000) with an increase in the cover percentage after 18 months (36%) compared to six months (23%) across all treatments. Coverage at both of these time periods was still less than the original measure (66%). However, this time effect was modified by several treatments as indicated by the following significant interactions with time: time*pine oil (p = 0.000); time*glyphosate (p<0.001); and time*seeding (p = 0.002). The analysis of the interactions indicated that pine oil and glyphosate were very effective after six months but not so effective after 18 months. However, whilst seeding is not as effective at six months, the effect lasts longer and is effective at 18 months.

The main treatments of pine oil (p = 0.000), glyphosate (p = 0.000) and seeding (p = 0.003) resulted in significant reductions in the measured cover. However, these need to be interpreted in context of the above time interactions and the pine oil*glyphosate interaction (p = 0.001). This interaction indicates that one or other treatment needs to be applied, but there was no improvement when applying both. Consideration of these results suggests that either pine oil or glyphosate should be used with seeding, and it is clear that the addition of seeding was more effective for both pine oil and glyphosate after 18 months. The mean percentage covers of these treatments are shown in Table 1 below and indicate that the most effective of these treatments was glyphosate with seeding across both time periods.

**Table 1:** Percentage cover of *Galenia pubescens* at six and 18 months for significant treatments.

Treatment	Six month cover %	18 month cover %
Control	62.0	66.5
Seeding	20.4	29.0
Glyphosate	15.1	32.7
Glyphosate + seeding	11.2	25.1
Pine Oil	14.7	33.3
Pine Oil + seeding	14.3	27.3
Average of all treatments	23.3	35.6

**Fig 1:**
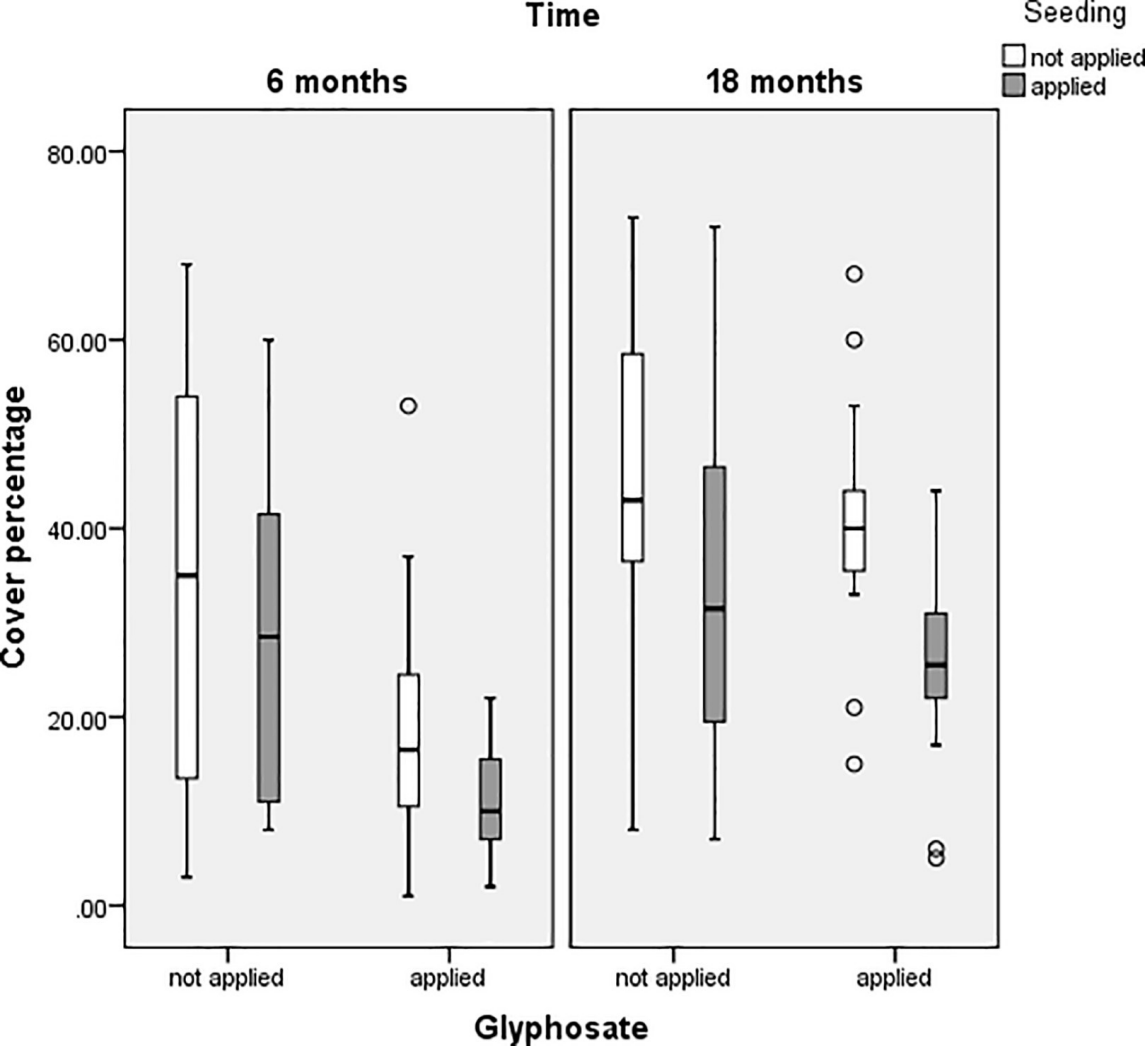
Boxplots comparing percentage cover of *Galenia pubescens* plots with or without application of glyphosate and seeding treatments.

Fig 1 shows the boxplots of the optimal treatments to reduce the cover percentage over six and 18 months. It can be seen that the application of Glyphosate is effective at six months and the addition of seeding produces more consistent results. Glyphosate alone does no better than the other treatments at 18 months, but the addition of seeding reduces the cover percentage and again produces more consistent results.

### *Galenia pubescens* seedbank abundance

The assumption checking indicated that the repeated measures analysis of seedbank abundance required a square-root transformation. This transformation did not quite meet the multivariate normality assumption, but was a marked improvement compared to the analysis of the original data. Further analysis of the problem associated with the multivariate normality assumption revealed that it appeared due to nonzero kurtosis rather than nonzero skew, and therefore is more robust to the departure of normality for the analysis conducted. The initial seedbank abundance was the covariate measure and was not significant (p = 0.342).

For seedbank abundance, the time effect was not significant (p = 0.165); however there were a couple of interactions with time: time*glyphosate (p = 0.008) and time*seeding (p = 0.016). Both interactions indicated that the application of glyphosate and seeding were more beneficial after 18 months compared to six months. There was also a significant three-way interaction of time*pine oil*mulch (p = 0.031). This interaction showed that when not using pine oil, the benefits of mulching were larger at six months compared to 18 months. There was slight improvement when using both pine and mulching at both time periods.

The main treatments of pine oil (p<0.001), mulch (p<0.00), and seeding (p = 0.023) were all significant. However, these need to be interpreted with the following interactions: pine oil*mulch (p<0.001); glyphosate*mulch (p = 0.019); and seed*mulch (p = 0.003). The seed*mulch interaction showed that one or the other treatment should be used but there was no benefit in using both. The glyphosate*mulch interaction indicated that it is better to use mulch alone, with no benefit being observed on addition of glyphosate. The pine oil*mulch interaction showed that while it was beneficial to use both materials, it was particularly useful to add mulch when not using pine oil.

The above results show that mulch and pine oil were the most significant main effects and there was no benefit with using seeding with mulch, but there was benefit in using pine oil with mulch (particularly at 18 months). The following Table 2 shows the mean seed abundance for the main treatments. The most effective of these treatments is mulch and pine oil together across both time periods.

**Table 2:** Seedbank abundance of *Galenia pubescens* at six and 18 months for significant treatments.

Treatment	Six month seed^0.5	18 month seed^0.5
Control	8.80	9.25
Mulch	1.65	3.20
Pine oil	1.87	3.37
Mulch + pine oil	1.22	2.66
Average of all treatments	3.23	4.40

**Fig 2:**
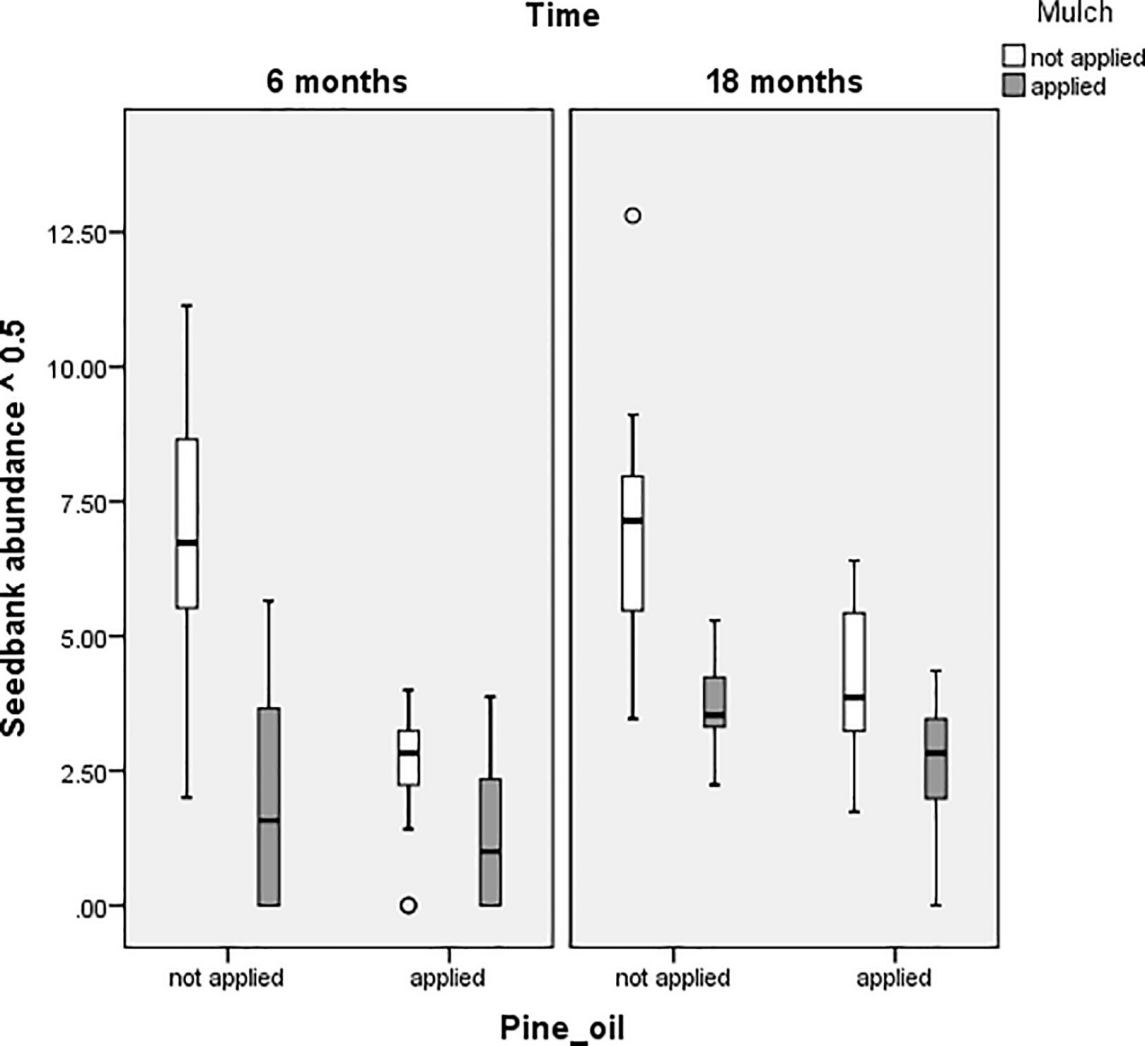
Boxplots comparing seedbank of *Galenia pubescens* abundance of plots with or without application of mulch and pine oil treatments.

Fig 2 shows the effectiveness in reducing seedbank abundance when applying mulch at both six and 18 months. The addition of pine oil provides extra benefit at 18 months.

### Common treatment for cover percentage and seedbank abundance

The previous sections identified the optimal treatments for both cover percentage (glyphosate and seeding) and seedbank abundance (pine oil and mulch). Ideally, a common treatment strategy should be applied to reduce both seedbank abundance and cover percentage. This section identifies a common treatment strategy for both cover percentage and seedbank abundance that produces results similar to the optimum treatments.

The statistical analyses indicate that pine oil and seeding are the only main effects that are significant in both cover percentage and seedbank abundance. Glyphosate and seeding was shown to be the optimal treatments for cover percentage. Pine oil can replace glyphosate as a treatment because pine oil also has a significant main effect (p<0.001) and the glyphosate*pine oil interaction (p = 0.001) shows that one or other should be used but not both together. Hence, pine oil can replace glyphosate, and together with seeding can have a minimal increase in the cover percentage compared to the optimal treatment. This is supported by the percentage covers shown in Table 1.

**Fig 3:**
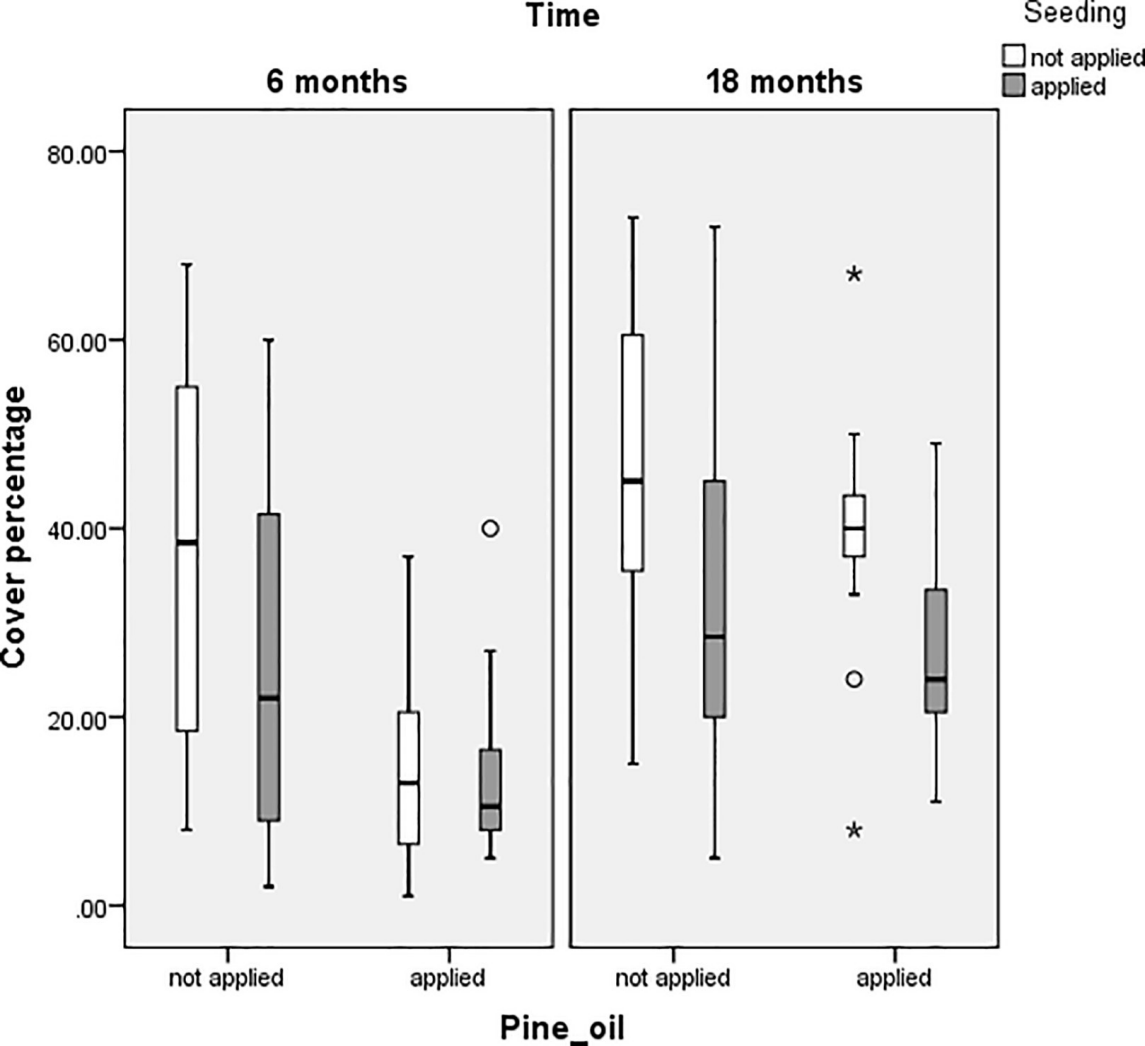
Boxplots comparing cover percentage of plots with or without application of pine oil and seeding treatments.

**Fig 4:**
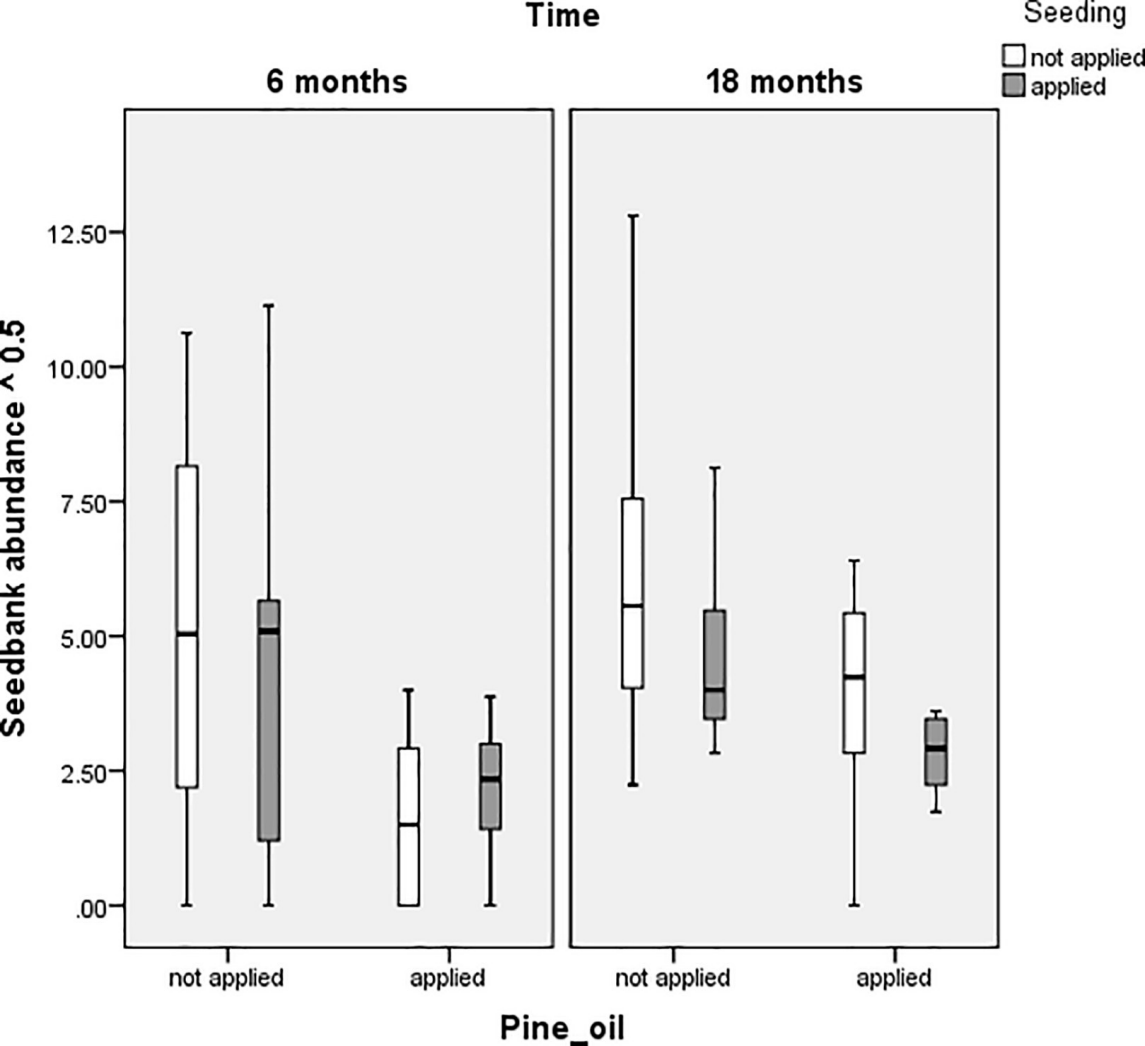
Boxplots comparing seedbank abundance of plots with or without application of pine oil and seeding treatments.

In terms of seedbank abundance, pine oil and mulch together produced the best results. Seeding can replace mulch as a treatment because seeding also has a significant main effect (p = 0.023) and the seed*mulch interaction (p<0.001) shows that one or other should be used, but not both together. This all indicates that pine oil and seeding is just as effective as pine oil and mulch in terms of reducing seedbank abundance. Figs 3 and 4 show the effects of applying pine oil and seeding to cover percentage and seedbank abundance at six and 18 months. The similarity of the results when applying pine oil and seeding compared to the optimal treatments are supported when comparing Figs 3 and 4 to Figs 1 and 2.

## Discussion

It is not unusual for plant populations to fluctuate with time and environmental change, and on occasions, elevated populations that have become a problem may require appropriate control management strategies. Often, allowing sufficient time for natural succession to be asserted can overcome this problem, but it has been suggested that this ‘do nothing’ approach may not be appropriate for all elevated populations of non-native species. One such example requiring intervention strategies is the infestation of *G*. *pubescens* in the Victorian grassland communities. As a consequence, the study presented here was aimed at assessing the effectiveness of four different intervention attempts to control *G*. *pubescens*: herbicide control with glyphosate; organic herbicide control with pine oil; the application of mulch; and addition of seeds of native species to the seedbank. In addition, all possible combinations of these techniques were tested. The management techniques examined in this study had a significant effect on both the cover percentage and number of viable *G*. *pubescens* seeds in the soil seedbank at six and 18 months after application of the various treatments, but the complexity of the treatment results indicates that the development of optimal management approaches needs to be carefully considered.

Regarding the effect of time of observation upon the changes to cover percentage and composition of the soil seedbank, the outcomes clearly showed that all the treatments tested showed some measure of success when measured six months after the date of application as compared to the untreated plots. However, the effectiveness of the treatments declined by 18 months, implying that in situations of heavy weed infestation a single round of control strategies is unlikely to be sufficient to kill all the mature plants or significantly deplete seed numbers. *G*. *pubescens* plants are capable of producing large numbers of seeds annually and of re-establishing themselves from undamaged parts of a treated plant [35].

Regarding the effectiveness of combinations of the treatments on reducing foliage cover percentage and density or quantity of soil seedbank, single treatments, as well as combinations of two, three or four treatments, were significantly effective within a few months. However, not all treatments were equally effective. Single treatments (for example pine oil or mulch) were less effective than when treatments were used in combinations of two, three or four. A combination of treatments is therefore recommended because it is likely to be more cost effective in the long-term [39, 40].

A study of another invasive weed species, perennial pepperweed (*Lepidium latifolium*), found that greater control was achieved when different treatments were combined, rather than single treatments being applied separately [41]. While our study was conducted over 18 months, its relevance to longer term control may perhaps be extrapolated from this experience. We assert that such management advice, if followed carefully, will significantly reduce the dominance of *G*. *pubescens* in affected areas. Pine oil application was the novel method used in this experiment, and this is the first time it has been used directly in the field for the control of a weed soil seedbank. The results we obtained show that this treatment is effective as a means to reduce the density of *G*. *pubescens* seedlings. Previous studies in pot trials had indicated that essential oils, such as pine oil, destroy seed viability by ‘exploding’ the outer coat of the seeds, causing cell damage and desiccation [42, 11]. In addition, Kremer and Spencer [43] stated that mechanical degradation of seeds, particularly from damage to the seed coat, makes the seeds more susceptible to attack by microorganisms. This suggests that microbial attack may have been partly responsible for the reduction in seedbank germination observed for the pine oil treatment in this trial. The changes in the seedbank composition of *G*. *pubescens* thus demonstrate that pine oil applications may have an important role in the control of weed seedbank for biodiversity conservation. The results indicate that if applications of pine oil are made regularly in areas of heavy weed infestation, a significant shift in seedbank composition will be achieved. Further, if the pine oil treatment is followed by an application of native plant seeds after a suitable withholding period, then the control will be more effective. The increased native plant population acts to reduce the incidence of bare soil, allows them to compete directly with weed re-establishment, and rapidly decreases the possibility of soil erosion. Weeds often spread at the expense of other species because they have ecological characteristics that enable them to reproduce and proliferate quickly. In general, the most aggressive weeds are characterised by their ability to spread rapidly beyond their natural distribution, high growth rates, rapid seedling recruitment, and high seed production, which suggests that management approaches need to be instituted with these attributes in mind.

In the case of glyphosate application, this non-selective herbicide was the only established chemical method used either independently or in combination to reduce foliage cover and seedbank density. It was found that this treatment was most effective in the short-term in reducing foliage cover of mature plants. There was some regrowth six months after the glyphosate treatment and further recovery by 18 months, although the total foliage cover was significantly reduced at each post-treatment time, compared to the control plots. This finding is in line with Cook [13], who showed that glyphosate application was relatively ineffective for control of *G*. *pubescens* if applied in spring, but moderately effective if applied in early and mid-autumn. In this study, it was suggested that the poor efficacy of application in spring was compounded by early signs of moisture stress, thus this ‘timing’ effect needs to be re-investigated without the interference of such secondary effects. The findings of Cook [13] support the continued use of autumn application of glyphosate, in combination with other treatments, to reduce infestations of *G*. *pubescens*.

The other effective method found in this study to control *G*. *pubescens* was mulch, a mixture of white sugar and sawdust. This treatment had a positive effect in reducing the cover abundance as well as retarding the seedling emergence of *G*. *pubescens*. There are a number of plausible explanations for the observed effects. Mulch forms a layer on the ground and prevents airborne or dropped seeds from reaching the soil surface, whilst also smothering mature plants and reducing their growth. Previous studies have also found that mulches are an effective technique to control weeds [44, 45, 46, 47]. Another advantage of incorporating sugar into a mulch mixture is promotion of the activity of microorganisms in the soil. Microorganisms, such as soil fungi, absorb soil nutrients as they grow, thereby reducing nutrient availability to weeds [48, 49]. This relative lack of nutrients inhibits or reduces weed growth, while simultaneously providing a better soil environment for native plants, which prefer low nutrient conditions [50]. In addition, certain organic mulches, such as sawdust or wood chips, may control weeds chemically through the leaching of naturally occurring allelopathic chemicals [51]. In a similar study, Greenly and Rakow [52] stated that organic mulches are most effective in weed control when applied to a sufficient depth, for example, so that they prevent light reaching the soil surface. Mulch treatment reduces light availability, which stresses existing weeds and prevents germination of their seeds, especially those with small seeds, such as *G*. *pubescens*.

Previous studies have shown that seeding alone is not a recommended solution to weed infestations [53, 54], since this treatment by itself does not usually result in an increased density of native species. It is possible that a larger increase in native plant cover may have been obtained if the introduction of the native seeds into the degraded area was made at a later time following the other treatments, (for example, one to three years after herbicide or mulch treatments, instead of simultaneously combined with the other treatments). This may have allowed time for the various combined treatments of herbicide, mulch or pine oil to achieve a greater reduction in seed bank weed seed density prior to re-seeding, thereby reducing potential competition of the weed with native species. Other authors have suggest that for ecosystems in a highly degraded state, rehabilitation efforts should first focus on removing the infestation of invasive species by the use of herbicides or biological controls [55, 56]. Once these interventions have significantly reduced weed cover density, direct seeding with the seeds of indigenous plants should take place [57, 58].

One of the most challenging issues in natural resource management is the control of elevated or unbalanced populations of introduced species into native vegetation communities and degraded land. Although substantial financial and other resources been directed towards the control of *G*. *pubescens*, natural resource management agencies in Victoria still struggle to manage the elevated populations. In this project we attempted to develop a suitable control method for this aggressive invasive species at landscape level. It can be concluded that application of treatments were most effective in reducing the population of *G*. *pubescens* when applied in combinations of multiple treatments. While our results did not assess the management costs associated with these various control practices, it is strongly suggested that a suitable combination of treatments would provide an ecologically and economically effective approach for control of *G*. *pubescens* over the long term, and therefore this approach should be further researched for the control of other perennial invasive weedy species.

## Supporting information

S1 File (CSV)

S2 File (CSV)
